# Identification and external validation of a novel miRNA signature for lymph node metastasis prediction in submucosal‐invasive gastric cancer patients

**DOI:** 10.1002/cam4.2530

**Published:** 2019-09-04

**Authors:** Mingzhe Ma, Shixun Lu, Yinhua Liu, Pengfei Kong, Ziwen Long, Ping Wan, Yan Zhang, Yanong Wang, Dazhi Xu

**Affiliations:** ^1^ Department of Gastric Surgery Fudan University Shanghai Cancer Center Shanghai China; ^2^ Department of Oncology Shanghai Medical College Fudan University Shanghai China; ^3^ Department of Pathology Sun Yat‐sen University Cancer Center Sun Yat‐sen University Guangzhou China; ^4^ Department of Pathology Yijishan Hospital The First Affiliated Hospital of Wannan Medical College Wuhu China; ^5^ Department of Liver Surgery Renji Hospital School of Medicine Shanghai Jiaotong University Shanghai China; ^6^ Department of Gastroenterology Yijishan Hospital The First Affiliated Hospital of Wannan Medical College Wuhu China

**Keywords:** gastric cancer, lymph node metastasis, miRNA, risk‐stratification

## Abstract

Endoscopic resection (ER) has been increasingly performed in the treatment of early gastric cancer (GC). However, lymph node metastasis (LNM) can cause treatment failure with ER, especially in T1b patients. Here, we attempted to develop a miRNA‐based classifier to detect LNM in T1b patients. Based on high‐throughput data from The Cancer Genome Atlas, we identified 20 miRNAs whose expression significantly changed in T1‐2 GC with LNM vs T1‐2 GC without LNM. We then developed a miRNA signature to predict LNM of T1b GC using the LASSO model and backward step wise elimination approach in a training cohort. Furthermore, the predictive accuracy of this classifier was validated in both an internal testing group of 63 patients and an external independent group of 114 patients. This systematic and comprehensive in silico study identified a 7‐miRNA signature with an area under the receiver operating characteristic curve (AUROC) value of 0.843 in T1‐2 GC and 0.911 in T1 EGC. The backward elimination was further used to develop a 4‐miRNA (miR‐153‐3p, miR‐708, miR‐940 and miR‐375) risk‐stratification model in the training cohort with an AUROC value of 0.898 in cohort 2. When pathologic results were used as a reference, the risk model yielded AUROC values of 0.829 and 0.792 in two cohorts of endoscopic biopsy specimens. This novel miRNA‐LNM classifier works better than the currently used pathologic criteria of ER in T1b EGC. This classifier could individualize the management of T1b patients and facilitate treatment decisions.

## Graphical abstract

The 4‐miRNA risk‐stratification model demonstrated higher sensitivity than the current pathologic criteria. The risk‐stratification model is of great clinical value in directing personalized treatment regimens. This model can identify true candidates for endoscopic resection in T1b GC patients, avoiding unnecessary surgery and reducing patients’ physical and economic burden

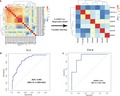

## 1 BACKGROUND

Gastric cancer (GC) is the fifth most prevalent cancer in regard to incidence and the fourth most common cause of cancer‐related death worldwide.^1^, ^2^ Early gastric carcinoma is defined as a malignant epithelial lesion of the stomach that is confined to the mucosa (T1a) or submucosa (T1b), irrespective of regional lymph node metastasis (LNM).^3^ Due to the mass population screening program in East Asia, up to 70% of GC are diagnosed as EGC.^4^

Endoscopic resection (ER), including endoscopic mucosal resection (EMR) and endoscopic submucosal dissection (ESD), has been used as the first‐line treatment for EGC with negligible risk of LNM.^4^ ER carries the benefit of minimal effects on patient quality of life, lower risks of complications from gastrectomy and similar long‐term outcomes to radical surgery.^5^, ^6^, ^7^, ^8^

LNM can result in ER treatment failure, since ER does not include lymph node dissection. Therefore, careful selection remains vital to avoid use of ER in patients with a high‐risk of LNM. Currently, various imaging techniques have been developed to predict nodal involvement, yet none of these techniques (including computed tomography (CT), endoscopic ultrasound (EUS), positron emission tomography, and magnetic resonance imaging) could meet the requirements of a high detection rate of infiltrated lymph nodes and a low frequency of false‐positive results, especially in EGC.^9^ Spolverato et al^10^ reported that tumor stage based on EUS often did not correlate with T stage or N stage on final pathologic analysis and 17% of patients have a risk of being misclassified as having N0 disease by preoperative EUS. A meta‐analysis concluded that EUS diagnostic performance cannot be considered to be optimal, especially in regard to the ability of EUS to distinguish T1a (mucosal) from T1b (submucosal) cancers and to identify positive versus negative lymph node status.^11^

Indeed, the prevalence of ER treatment failure is higher in T1b patients than in T1a patients, because submucosal‐invasive GC harbors a much higher LNM rate (19.2% for T1b vs 3.2% for T1a).^12^ Tremendous efforts have been put into the exploration of ER criteria for T1b. However, current pathologic criteria do not accurately predict the risk of LNM for patients with T1b GC. For example, LNM was noted in EGC patients who fulfilled the expanded criteria in submucosal‐invasive GC, as reported by Kang et al^12^ (LNM 3/20, 15.0%) and Hanada et al^13^ (LNM 1/4, 25.0%). Thus, novel, reliable and objective biomarkers should be identified to determine genuinely high‐risk patients for LNM in T1b GC.

MiRNAs are a class of non‐protein coding RNAs (18‐25 nucleotides in length) that regulate the degradation of messenger RNAs (mRNAs) via seed sequence base‐pairing.^14^ MiRNA profiles have been shown to be tissue and disease specific^15^ and thus can be used as biomarkers for the diagnosis and prediction of prognosis as well as treatment sensitivity in a variety of cancers.^16^, ^17^, ^18^, ^19^

Here, based on data from The Cancer Genome Atlas (TCGA), we performed a comprehensive study to identify multi‐miRNA‐based classifiers to detect LNM in T1b GC. Importantly, we validated the clinical significance of this classifier in multiple clinical cohorts, including endoscopy‐derived biopsy samples.

## 2 MATERIALS AND METHODS

### 2.1 Patient cohort

**Figure 1:**
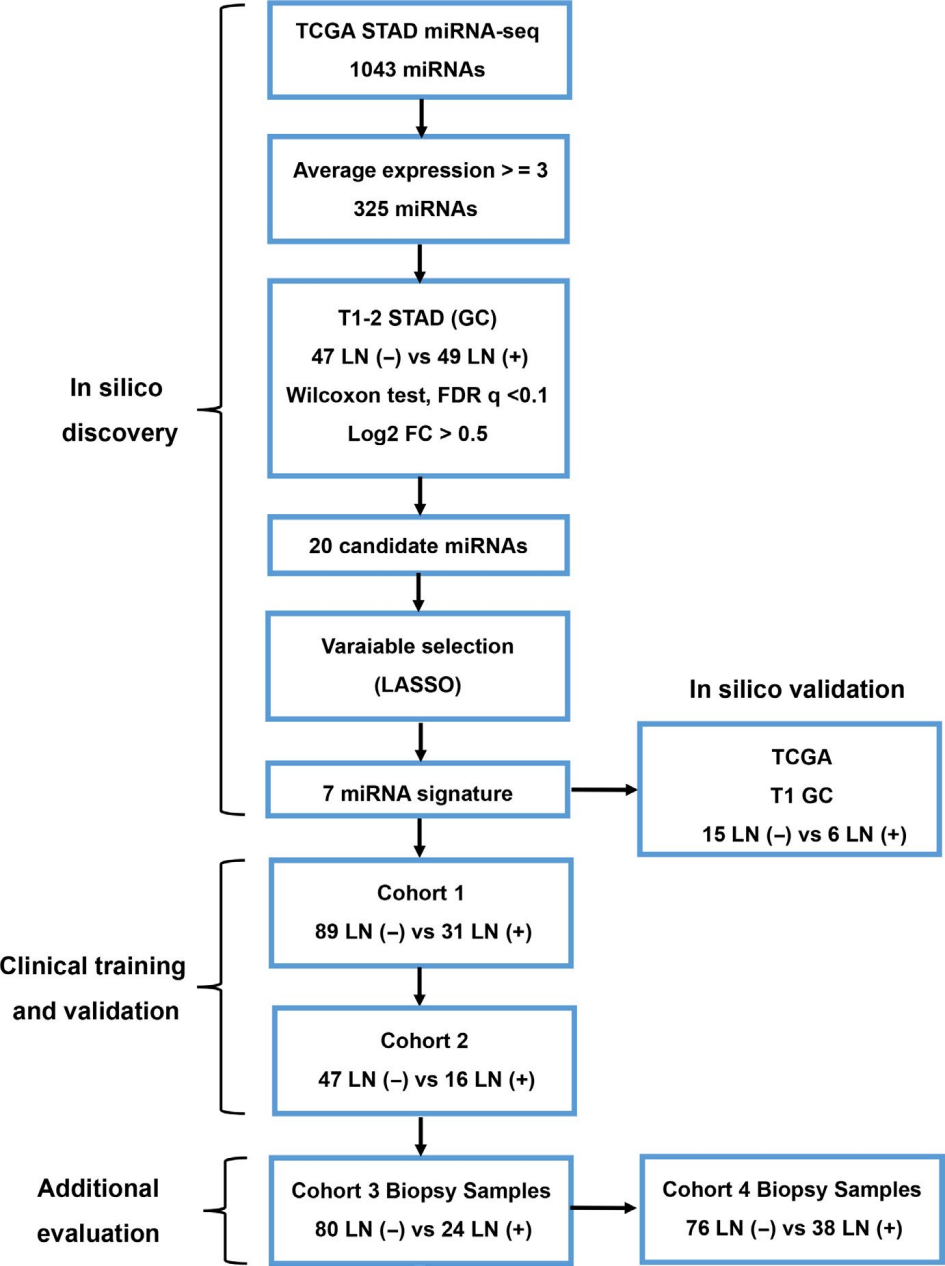
The flowchart of this study

The samples used in different parts of this study are summarized in Figure 1. This study included multiple clinical cohorts with a total of 393 GC patients. These patients included patients from the publicly available TCGA dataset (n = 96), as well as two cohorts of 297 T1b GC patients who did not receive any preoperative chemo‐ or radio‐therapy. The first cohort comprised 183 formalin‐fixed paraffin‐embedded (FFPE) specimens from patients who underwent curative D2 gastrectomy at the Yijishan Hospital, Wannan Medical College (Wuhu, Anhui, China) from 2014 to 2017. We randomly assigned approximately two‐thirds of the patients in this cohort to the training cohort (n = 120, cohort 1) for the construction of a miRNA signature and one‐third of the patients to the validation cohort (n = 63, cohort 2). Matched FFPE endoscopic biopsy samples from 104 patients (cohort 3, 72 from cohort 1 and 32 from cohort 2) were taken by gastroscopy prior to surgery. Another cohort of 114 FFPE specimens from 114 patients (cohort 4) were enrolled at Sun Yat‐sen University Cancer Center, Sun Yat‐sen University (Guangzhou, China) from 2012 to 2018 and were taken by gastroscopy prior to surgery. The exclusion criteria were as follows: age <18 years, presence of metastasis, nonadenocarcinoma, nonavailability of FFPE specimens or patient demographics, non‐EGC, presence of preoperative chemo‐ or radio‐therapy and non‐D2 gastrectomy. All samples were evaluated by two independent pathologists according to the 8th edition of the American Joint Committee on Cancer (AJCC) tumor‐node‐metastasis (TNM) staging system. In the pathological examination, tumors in which the percentages of undifferentiated‐type components ≥50% were deemed as undifferentiated GC. Data on patient demographic and clinicopathological features, including gender, age at surgical resection, tumor location, tumor size, macroscopic appearance, depth of invasion, number of positive lymph nodes, number of lymph nodes retrieved, lymphovascular invasion, tumor differentiation, preoperative serum carcinoembryonic antigen, carbohydrate antigen 72‐4, and carbohydrate antigen 19‐9 were collected. Computed tomography data collected prior to surgery were retrieved and evaluated by two independent radiologists, and any discrepancy between assessments was resolved by discussion or by a third radiologist. The study methodologies conformed to the standards set by the Declaration of Helsinki. Written consent was obtained from each subject and this study was approved by and performed under the censorship of the local ethics committee of each contributing center. The detailed clinicopathological characteristics are shown in Table 1.

**Table 1:** Clinicopathological Features of Cohort 1, 2, 3 and 4

Characteristics	Surgical specimens, n (%)		Biopsy specimens, n (%)	
	Cohort 1	Cohort 2	Cohort 3	Cohort 4
Gender				
Male	86 (71.67)	45 (71.43)	73 (70.19)	74 (64.91)
Female	34 (28.33)	18 (28.57)	31 (29.81)	40 (35.09)
Age (y)				
≤59	46 (38.33)	24 (38.10)	37 (35.58)	67 (58.77)
≥60	74 (61.67)	39 (61.90)	67 (64.42)	47 (41.23)
Tumor site				
Upper third	26 (21.67)	14 (22.22)	23 (22.11)	14 (12.28)
Middle third	14 (11.66)	8 (12.70)	14 (13.46)	41 (35.96)
Lower third	80 (66.67)	41 (65.08)	67 (64.42)	59 (51.76)
Tumor depth				
<500 µm	37 (30.83)	18 (28.57)	34 (32.69)	33 (28.95)
≥500 µm	83 (69.17)	45 (71.430	70 (67.31)	81 (71.05)
Lymph node metastasis				
Negative	89 (74.17)	47 (74.60)	80 (76.92)	76 (66.67)
Positive	31 (25.83)	16 (25.40)	24 (23.08)	38 (33.33)
Tumor size (cm)				
<3	85 (70.83)	44 (69.84)	77 (74.04)	79 (69.30)
≥3	35 (29.17)	19 (30.16)	27 (25.96)	35 (30.70)
Gross type				
Elevated/flat	82 (68.33)	40 (63.50)	65 (62.50)	72 (63.16)
Depressed	38 (31.67)	23 (36.50)	39 (37.50)	42 (36.84)
Histology				
Well and moderate	71 (59.17)	37 (58.73)	60 (57.69)	61 (53.51)
Poor	49 (40.83)	26 (41.27)	44 (42.31)	53 (46.49)
Lymphovascular tumor emboli				
Absent	99 (82.50)	51 (80.95)	86 (82.69)	93 (81.58)
Present	21 (17.50)	12 (19.05)	18 (17.31)	21 (18.42)
Lymph nodes retrieved				
<15	0 (0)	0 (0)	0 (0)	0 (0)
≥15	120 (100)	63 (100)	104 (100)	114 (100)
CEA				
Normal	104 (86.67)	54 (85.71)	88 (84.62)	97 (85.09)
High	16 (13.33)	9 (14.29)	16 (15.38)	17 (14.91)
CA19‐9				
Normal	108 (90.00)	58 (92.07)	93 (89.42)	101 (88.60)
High	12 (10.00)	5 (7.93)	11 (10.58)	13 (11.40)
CA72‐4				
Normal	103 (85.83)	52 (82.54)	92 (88.46)	98 (85.96)
High	17 (14.17)	11 (17.46)	12 (11.54)	16 (14.04)
CT diagnosis				
Lymph node‐negative	84 (70.00)	44 (69.84)	76 (73.08)	71 (62.28)
Lymph node‐positive	36 (30.00)	19 (30.16)	28 (26.92)	43 (37.72)

Abbreviations: CEA, carcinoembryonic antigen; CT, computed tomography.

### 2.2 Candidate miRNA selection and miRNA signature identification using TCGA data

TCGA level‐3 miRNA expression data for GC were downloaded from the Firehose Broad GDAC portal (accessed on 13 March 2018). The acquired dataset contained expression data from 1043 noted miRNAs. The miRNA expression levels, measured by reads per million (RPM) for each miRNA mapped, were log2 transformed. First, the miRNA expression levels between LNM (+) and LNM (−) samples in T1‐2 GC samples (n = 93; 48 LNM (+) and 45 LNM (−)) were compared utilizing the following criteria: absolute log2‐fold‐change >0.5; false discovery rate (FDR) q < 0.1; Wilcoxon rank‐sum test *P* < .01; and relatively high expression levels of miRNA (count per million >3).

### 2.3 RNA isolation, cDNA biosynthesis and quantitative real‐time polymerase chain reaction (qRT‐PCR)

Total RNA was extracted from 10‐μm‐thick FFPE specimens utilizing an AllPrep DNA/RNA FFPE kit (Qiagen), following the manufacturer's instructions. Complementary DNA was synthesized with miRNA‐specific Bugle‐Loop primers (RiboBio) and an M‐MLV RT kit (Invitrogen). Real‐time RT‐PCR was performed using an ABI 7500 sequence detection system (Applied Biosystems). The relative expression of miRNAs was calculated by the 2^−ΔCt^ method using small nuclear RNA U6 as an internal control. The normalized values were log10 transformed. The primers used in this study were purchased from RiboBio. We observed no difference in U6 expression between LNM (+) and LNM (−) patients. The real‐time PCRs were performed in triplicate.

### 2.4 Statistical analysis

Data are expressed as the mean ± standard deviation (SD) from three independent replicates. All statistical analyses were performed using IBM SPSS version 17, GraphPad Prism version 5.0 and R software 3.4.0. Unpaired Student's *t* test was used to determine the difference in miRNA expression levels between LNM (+) and LNM (−). Statistical differences of various clinicopathological factors between LNM (+) and LNM (−) patients were determined with Pearson's χ^2^ test for categorical data. Pearson's correlation coefficient was used for the expression correlation assay. Receiver operating characteristic (ROC) curves were generated to distinguish GC patients with and without LNM. Predictive accuracy was determined by measuring the area under the ROC curve (AUROC), specificity and sensitivity. A predictive model with an AUROC of >0.7 was considered to be sufficiently discriminative. The stepwise backward regression was used for miRNA selection. As all of the miRNAs selected fulfilled the criteria of AUROC >0.7 in the individual analyses, we trained a classifier based on four miRNAs with binary logistic regression. The risk score was calculated using a formula derived from the training cohort: Risk Score = 6.001619 × miR‐153‐3p + 4.454248 × miR‐708 + 1.971937 × miR‐940 + 5.111626 × miR‐375 + 35.399131. The weights and cutoff thresholds derived from the training cohort were used in the validation cohort. All *P*‐values are two‐sided and a *P*‐value less than .05 was considered to be statistically significant.

## 3 RESULTS

### 3.1 Identification of LNM‐specific miRNAs by analyzing TCGA dataset

**Figure 2:**
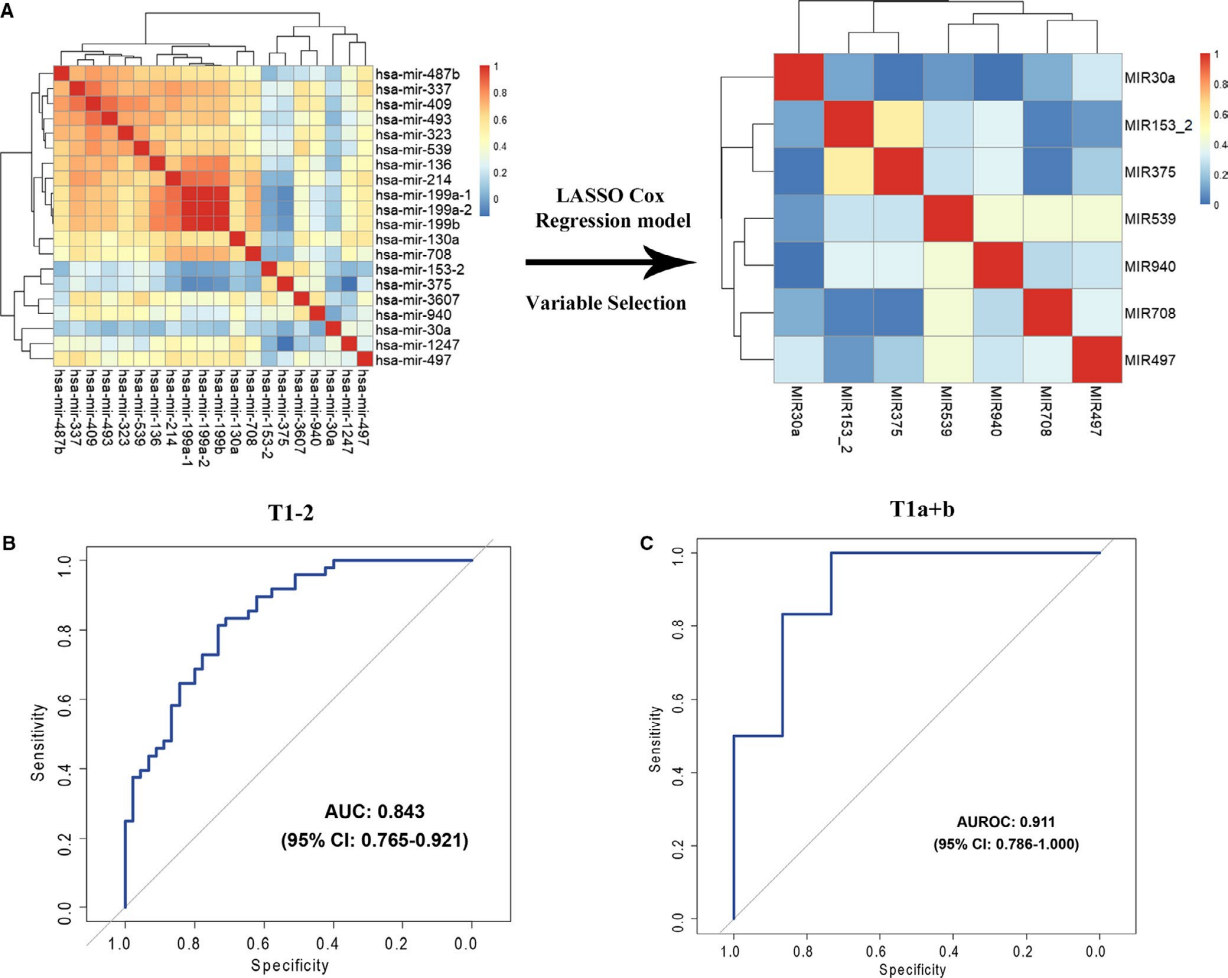
Variable selection and in silico validation. A, Hierarchical clustering shows the collinearity of 20 candidate miRNAs. Correlation matrix heatmap of 20 miRNAs in the training cohort, where each cell represents the Pearson correlation between the row and column corresponding miRNAs. The legend shows the color change along with the change of correlation coefficient from 0 to 1.0. LASSO Cox regression model to select miRNAs to predict the LNM of the patients in the TCGA dataset, which yielded seven miRNAs. B, The 7‐miRNA signature showed an AUROC of 0.843 (95% confidence interval [CI], 0.765‐0.921) to discriminate LNM‐positive (n = 49) and LNM‐negative (n = 47) in T1‐2 GCs, and (C) an AUROC of 0.911 (95% CI, 0.786‐1.000) to discriminate LNM‐positive (n = 6) and negative (n = 15) in T1 GCs

The study design is illustrated in Figure 1. We used TCGA dataset as the discovery cohort and compared the miRNA expression profiles between LNM (+) and LNM (−) T1‐2 GC patients. We established 20 miRNAs with an absolute log2‐fold‐change >0.5, FDR q < 0.1, *P* < .01 and an average expression level ≥3 transcripts per million (Figure 2A). To further validate the in silico findings, we analyzed the expression of the 20 miRNAs in T1b GC from TCGA (12 LNM (−) vs 6 LNM (+), Figure S1) and cohort 1 (10 LNM (−) vs 10 LNM (+), Figure S2). The in silico and qRT‐PCR validation confirmed the findings from TCGA, indicating that a set of miRNAs are frequently dysregulated in T1b LNM (+) patients. However, we observed some collinearity among some miRNAs, which could prejudice the results. Therefore, we used the LASSO Cox regression model to select miRNAs and established a signature with a panel of seven miRNAs (miR‐153‐3p, miR‐30a, miR‐539, miR‐708, miR‐940, miR‐497 and miR‐375, Figure 2A, Figure S3). A miRNA signature based on the expression of the seven miRNAs yielded an area under the receiver operating characteristic curve (AUROC) of 0.843 for predicting LNM in T1‐2 GC patients from TCGA (n = 96, 47 LNM (−) vs 49 LNM (+), Figure 2B). The AUROC values for predicting LNM in T1 GC patients (T1a + T1b, n = 21, 15 LNM (−) vs 6 LNM (+), Figure 2C, Figure S4), T1b patients (n = 18, 12 LNM (−) vs 6 LNM (+), Figure S5) were 0.911 and 1.000 (data not shown), respectively. These AUROC values highlight the validity of the miRNA signature.

### 3.2 Further selection and establishment of the miRNA signature

**Figure 3:**
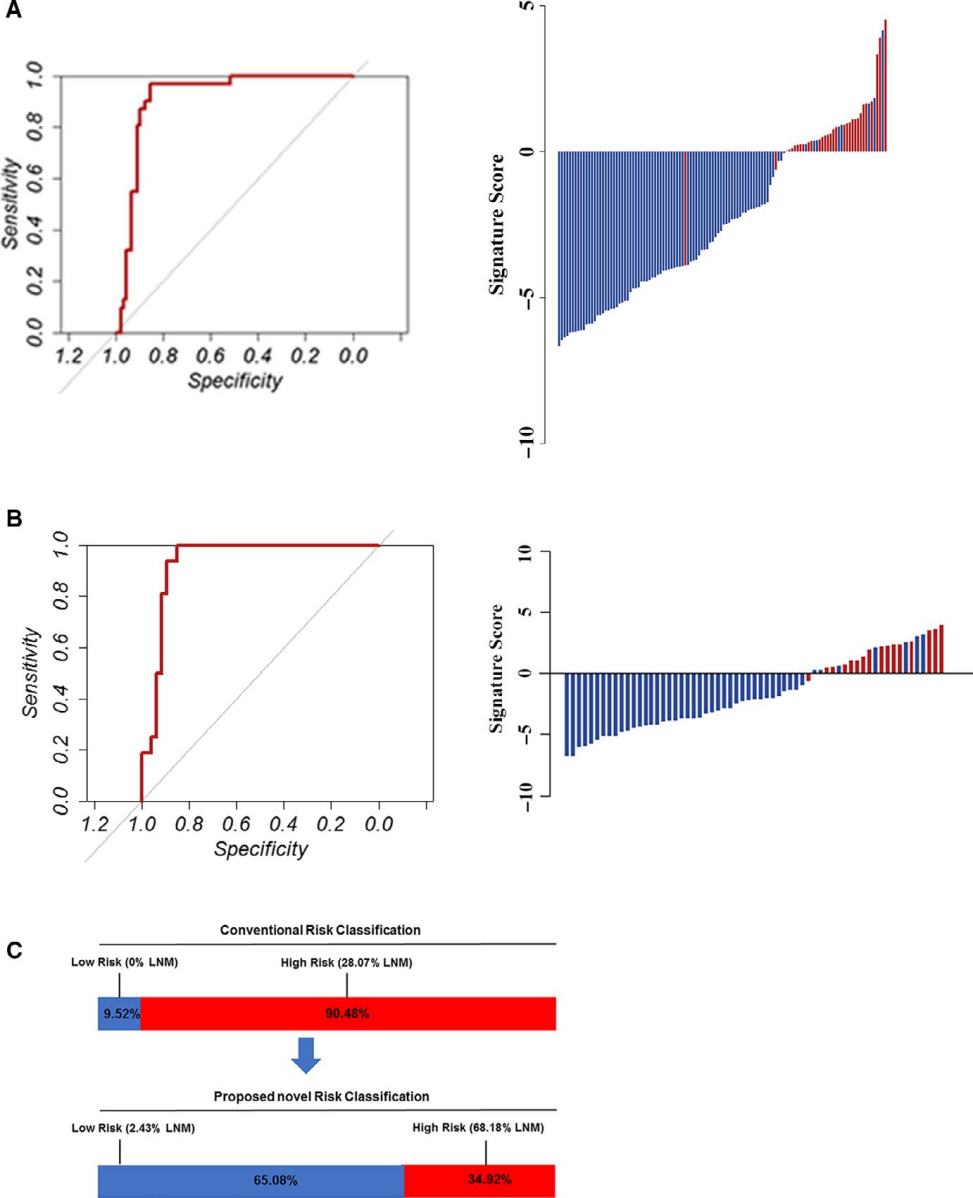
Clinical model training and validation. A and B, The detection values of the 4‐microRNA (miRNA) signature in each patient (red line: positive for LNM, blue line: negative for LNM). The cutoff threshold was set as −0.9. The 4‐miRNA signature revealed AUROC values of 0.950 in the training cohort (A) and 0.938 in the validation cohort (B) for discriminating LNM‐positive and LNM‐negative patients. C, According to the conventional pathologic criteria to predict LNM, 9.52% patients were classified into the low‐risk group (0% LNM) and 90.48% patients into the high‐risk group (28.07% LNM). However, the novel risk‐classification model identified 34.92% as high‐risk (68.18% LNM) and 65.08% as low‐risk (2.43% LNM)

To test whether our finding from the in silico datasets could be applied in clinical settings, we measured the expression levels of seven miRNAs in 120 FFPE specimens (cohort 1) and developed a risk score formula to predict LNM. Detailed clinicopathological characteristics are shown in Table 1. Associations between LNM and clinicopathological features are shown in Table S1. A backward stepwise elimination approach was applied and identified four miRNAs (miR‐153‐3p, miR‐708, miR‐940, and miR‐375) for the development of a risk‐classification model. The four identified miRNAs all yielded an AUROC value >0.7 in the in silico datasets (T1b GCs, Figure S5) and >0.8 in the training cohort (Figure S6). The following risk score formula was developed: risk score = 6.001619 × miR‐153‐3p + 4.454248 × miR‐708 + 1.971937 × miR‐940 + 5.111626 × miR‐375 + 35.399131. The predicted risks of all patients were calculated with this formula. The 4‐miRNA signature achieved an impressive AUROC value of 0.872 (95% CI: 0.823‐0.918) in the training cohort (Figure 3A). To evaluate the robustness of the risk‐classification model, we examined its performance in the validation cohort. The risk‐classification model achieved excellent risk stratification in the validation cohort (AUROC = 0.898, 95% CI: 0.866‐0.959) (Figure 3B). According to the conventional pathologic criteria that are used to predict LNM, 9.52% of patients were classified as the low‐risk group (0% LNM) and 90.48% of patients were classified as the high‐risk group (28.07% LNM). However, the novel risk‐classification model identified 34.92% as high‐risk (68.18% LNM) and 65.08% as low‐risk (2.43% LNM) (Figure 3C).

### 3.3 Validation of the miRNA classifier in endoscopic biopsy specimens to evaluate its translational potential

**Figure 4:**
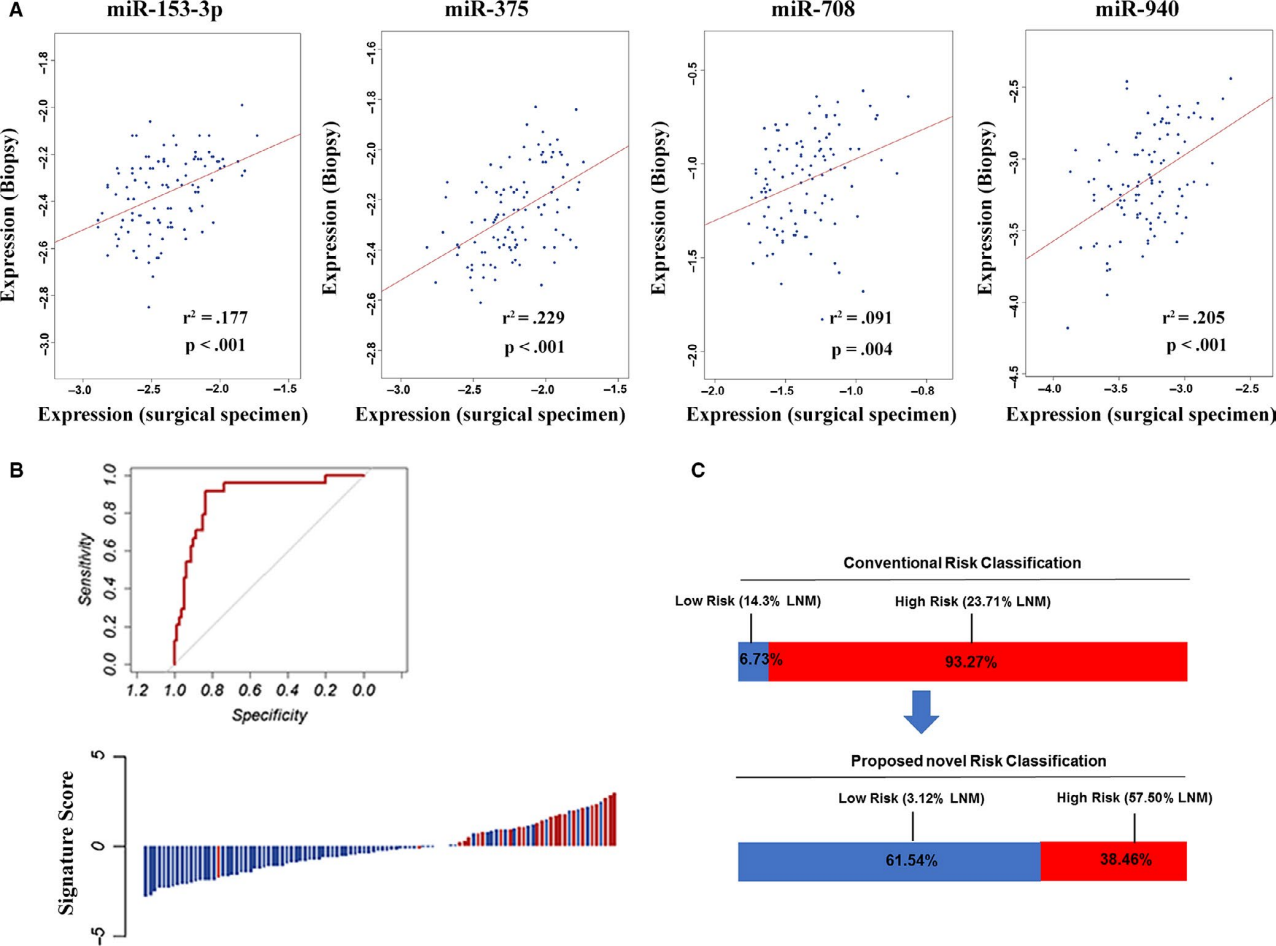
Validation in biopsy specimens. A, Pearson's correlation analyses of miRNA expression between the surgical and the biopsy specimens. The expression levels of miR‐153‐3p, miR‐708, miR‐940 and miR‐375 were significantly correlated between surgically resected and biopsy samples. B, The detection values of the 4‐microRNA (miRNA) signature in each patient in cohort 3 (red line: positive for LNM, blue line: negative for LNM). The 4‐miRNA signature revealed AUROC values of 0.907 in cohort 3 for discriminating LNM‐positive and LNM‐negative patients. C, According to the conventional pathologic criteria to predict LNM, 6.73% patients were classified into the low‐risk group (14.3% LNM) and 93.27% patients into the high‐risk group (23.71% LNM). However, the novel risk‐classification model identified 38.46% as high‐risk (57.50% LNM) and 61.54% as low‐risk (3.12% LNM)

To determine its clinical utility, we next assessed the predictive accuracy of the miRNA signature for LNM in 104 FFPE biopsy samples (cohort 3, 32 from cohort 1 and 72 from cohort 2) taken by gastroscopy prior to surgery (Table 1). The association among LNM and clinicopathological features in cohort 3 is shown in Table S2. The expression levels of the four miRNAs in endoscopic biopsy specimens were all significantly correlated with those of surgically resected samples (Figure 4A). We employed an independent logistic regression model to these endoscopic biopsy specimens and reached an AUROC value of 0.829, with a 95% CI of 0.753‐0.907, which suggests that the miRNA signature could accurately predict LNM in endoscopic biopsy specimens (Figure 4B,C).

**Figure 5:**
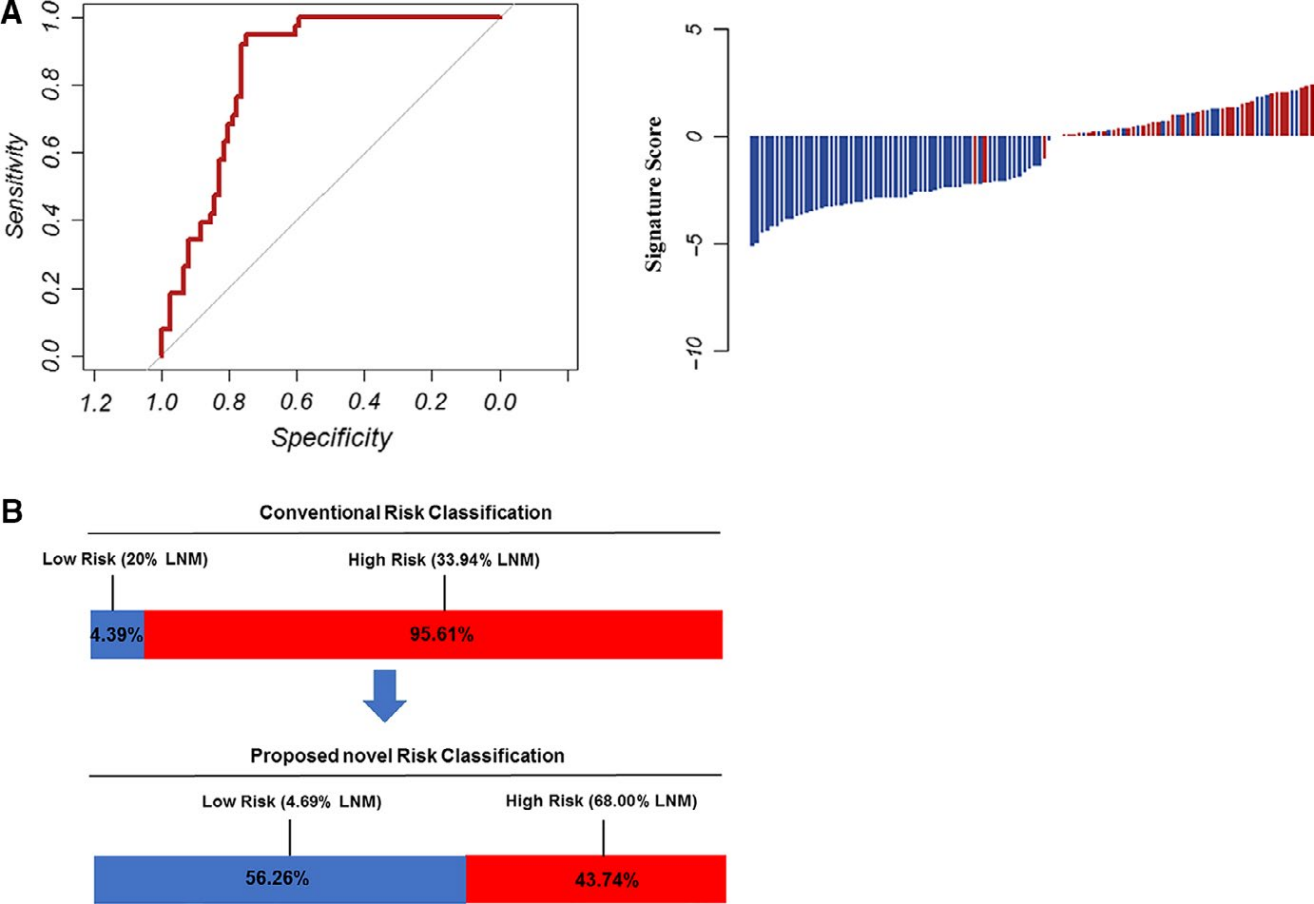
Additional validation in biopsy specimens. A, The detection values of the 4‐microRNA (miRNA) signature in each patient in cohort 4 (red line: positive for LNM, blue line: negative for LNM). The 4‐miRNA signature revealed AUROC values of 0.921 in cohort 4 for discriminating LNM‐positive and LNM‐negative patients. B, According to the conventional pathologic criteria to predict LNM, 4.39% patients were classified into the low‐risk group (20% LNM) and 95.61% patients into the high‐risk group (33.94% LNM). However, the novel risk‐classification model identified 43.74% as high‐risk (68.00% LNM) and 56.26% as low‐risk (4.69% LNM)

We assessed the predictive accuracy of the miRNA classifier in an additional cohort of 114 endoscopic biopsy specimens (FFPE) from the Sun Yat‐sen University Cancer Center at Sun Yat‐sen University in Guangzhou, China. As anticipated, the miRNA classifier yielded an AUROC value of 0.792, with a 95% CI of 0.731‐0.873, which further confirmed its translational potential (Figure 5A,B). Furthermore, we evaluated the survival significance of the miRNA signature with data from TCGA. We found that the miRNA signature could significantly predict the survival of GC patients (Figure S7).

## 4 DISCUSSION

In this study, we developed a 4‐miRNA (miR‐153‐3p, miR‐708, miR‐940 and miR‐375) LNM risk classifier in submucosal‐invasive GC patients that yielded an impressive predictive accuracy for lymph node metastasis. We further validated the LNM risk‐stratification model in two independent cohorts of endoscopic biopsy specimens.

MiRNAs have emerged as vital biomarkers due to their tumor and tissue specificity, their ability to resist RNase‐mediated degradation (possibly due to their short length) and their intact expression in FFPE tissues as well as in bodily fluids (including blood samples).^14^, ^17^ Two miRNA‐based models have been proposed to predict LNM in T1 colorectal cancer (CRC).^19^, ^20^ Jung et al^20^ established a three‐miRNA classifier (miR‐342‐3p, miR‐361‐3p, and miR‐3621) for predicting LNM in T1 stage CRC, reaching the area under the curve of 0.947. However, the unavailability of validation data in a large cohort (n = 20) and in endoscopic biopsy specimens limits its clinical value. Ozawa et al^19^ established another miRNA (miR‐32, miR‐181b, miR‐193b, miR‐195, and miR‐411) signature based LNM risk‐classification model to predict LNM in T1 stage CRC with in silico data that achieved an AUROC value of 0.77 in biopsy specimens. In view of the inconsistency between the two studies, we performed an unbiased, systematic and comprehensive genome‐wide analysis with data from TCGA to identify a robust miRNA classifier. Unexpectedly, the predictive accuracy of this model was quite impressive both in the internal and external validation cohorts.

Compared to the criteria of T1a patients, the criteria of ER for submucosa‐invasive (T1b) patients is more controversial because of the high risk of LNM in T1b.^21^ Gotoda et al^22^ and Park et al^23^ found that patients with one or more of the following factors have high‐risk of LNM: presence of lymphovascular emboli, depth of submucosal invasion ≥500 µm, tumor diameter ≥3 cm and undifferentiated histology. According to these studies, differentiated minute submucosal‐invasive (tumor invasion into the upper third of the submucosa, ≤500 µm, SM1) carcinoma with a diameter ≤3 cm can be accepted in the expanded criteria for ESD in T1b patients. Favorable long‐term outcomes have been demonstrated for lesions fulfilling either the standard or expanded criteria after ER.^8^ As the LNM prevalence is 3.2% and 19.2% in mucosal and submucosal EGC, respectively,^23^ the selection of patients is particularly important in T1b GC.

However, questions have been raised about the predictive power of the currently used pathologic features for LNM in T1b patients. First, with respect to the evaluation of lymphovascular emboli, which is the strongest predictor for LNM,^8^ there is debate about the recognition, diagnosis, and objectivity of lymphovascular emboli in cancers.^24^, ^25^, ^26^ Although immunohistochemical staining could yield better detection of lymphovascular emboli than conventional hematoxylin and eosin staining, additional prospective studies are warranted.^24^, ^27^ Second, concerning the depth of submucosal invasion, Cho et al^28^ argued that the maximal depth of submucosal invasion is inappropriate as the current cutoff value (500 µm) was determined from surgical specimens but not from endoscopically resected lesions. The thickness of the submucosa decreased after the specimen was stretched; thus a cutoff value less than 500 μm should be adopted. Differences in the methods of measurement, especially when the muscularis mucosa is irregular and partially effaced due to malignant infiltration and desmoplasia, could have a significant impact on the results.^25^, ^29^ Thus, the evaluation of lymphovascular emboli and depth of invasion can only be performed in specimens from ER, which is used to select suitable patients for further surgical intervention. It limits their practicality and efficacy. Finally, the evaluation of undifferentiated histology is especially difficult in GC. Greater histologic diversity is a well‐known characteristic of GC, which even presents in intramucosal cancers. The histologic diversity tends to increase with invasion depth and tumor diameter.^23^, ^30^ Moreover, it is difficult to evaluate the percentage of undifferentiated components with surface characteristics from endoscopic examination.^23^, ^31^

Disagreement also arises in regard to the maximal tumor diameter. Hölscher et al argued that ER is not indicated in submucosal‐invasive lesions with diameters ≥2 cm.^32^ Other studies have also demonstrated that a diameter of the tumor ≥2 cm was an independent predictor of lymph node metastasis in submucosal‐invasive GC.^33^, ^34^, ^35^ These results were in consistent with our findings in this study: tumor diameter ≥3 cm was not correlated with LNM in four cohorts of patients. More research should be conducted to determine the optimal cutoff value of tumor diameter in the Chinese population. In view of all of these studies, the validity of the currently used pathologic criteria of ER is disputed. Additionally, our data showed that in cohorts 3 and 4, LNM was observed in T1b EGCs, which fulfilled the current expanded pathologic criteria of ER. The current pathologic criteria also have low sensitivity compared to this novel risk‐stratification model. According to our study, approximately 90% of T1b GC patients can be classified as high‐risk; however, only approximately 25% of patients have LNM. All of these data suggest that novel risk‐stratification models should be proposed.

The predictive power of our risk‐stratification model is quite impressive, in view of the fact that tiny biopsy specimens do not always represent the intratumoral heterogeneity and could cause deviations.^14^ Our risk‐stratification model yielded higher sensitivity in the biopsy specimens from the Sun Yat‐sen University Cancer Center. This may be due to the high incidence of LNM in this cohort (33.33%), while the incidence of LNM was 25.83%, 25.40%, 23.08% in cohort 1, cohort 2, cohort 3, respectively. We hypothesize that the risk‐stratification model might work better in populations with higher LNM rates. The LNM prevalence in T1b patients is generally higher in Western patients than in Eastern populations.^12^, ^35^, ^36^ In a cohort of 67 EGC patients in the USA, LNM was present in 32% (14/44) of T1b tumors.^37^ A 2018 study of 176 EGC cases from the USA reported an LNM rate of 33.9%.^13^ Further research should be conducted to determine the predictive power of this risk‐stratification model in Western populations.

## 5 CONCLUSION

An ideal predictive model is vital for refining treatment selections and thereby improving the survival and quality of life of patients. We developed a four miRNA (miR‐153‐3p, miR‐708, miR‐940 and miR‐375)‐based LNM risk‐stratification model that manifested superior predictive accuracy than the currently used clinicopathological criteria of ER. Our findings may be of great clinical value in directing personalized treatment regimens. This model can identify true candidates for ER in T1b GC patients, avoiding unnecessary surgery and reducing patients’ physical and economic burden.

## CONFLICT OF INTERESTS

The authors declare no conflicts of interest.

## AUTHOR CONTRIBUTIONS

Conception and design: M.‐z. Ma, Y. Zhang, S.‐X. Lu, P. Wan. Development of methodology: M.‐z. Ma, Y. Zhang, Y.‐X. Liu. Acquisition of data: M.‐z. Ma, Y. Zhang, P.‐F. Kong, Z.‐W Long, Y. Zhang, Y.‐N. Wang. Analysis and interpretation of data (eg, statistical analysis, biostatistics, computational analysis): M.‐z. Ma, Y. Zhang, Z.‐W Long, Y. Zhang Writing, review, and/or revision of the manuscript: M.‐z. Ma, Y. Zhang, D.‐Z. Xu. Administrative, technical, or material support (ie, reporting or organizing data, constructing databases): M.‐z. Ma, D.‐Z. Xu. Study supervision: M.‐z. Ma, D.‐Z. Xu.

## ETHICS APPROVAL AND CONSENT TO PARTICIPATE

All authors approved and directly participated in the planning, execution and/or analysis of the data presented herein. Written consent was obtained from each subject and this study was approved by and performed under the censorship of the local ethics committee of each contributing center.

## Data Availability

The datasets used for the current study are available from the corresponding author on reasonable request.
