# Supplementary material for: Identification and external validation of a novel miRNA signature for lymph node metastasis prediction in submucosal‐invasive gastric cancer patients
Source: Cancer Med. 2019 Sep 4;8(14):6315–25. doi: 10.1002/cam4.2530 (PMC6797584; doi:10.1002/cam4.2530)
Supplement: Supplementary file 1 [file CAM4-8-6315-s001.docx]

**
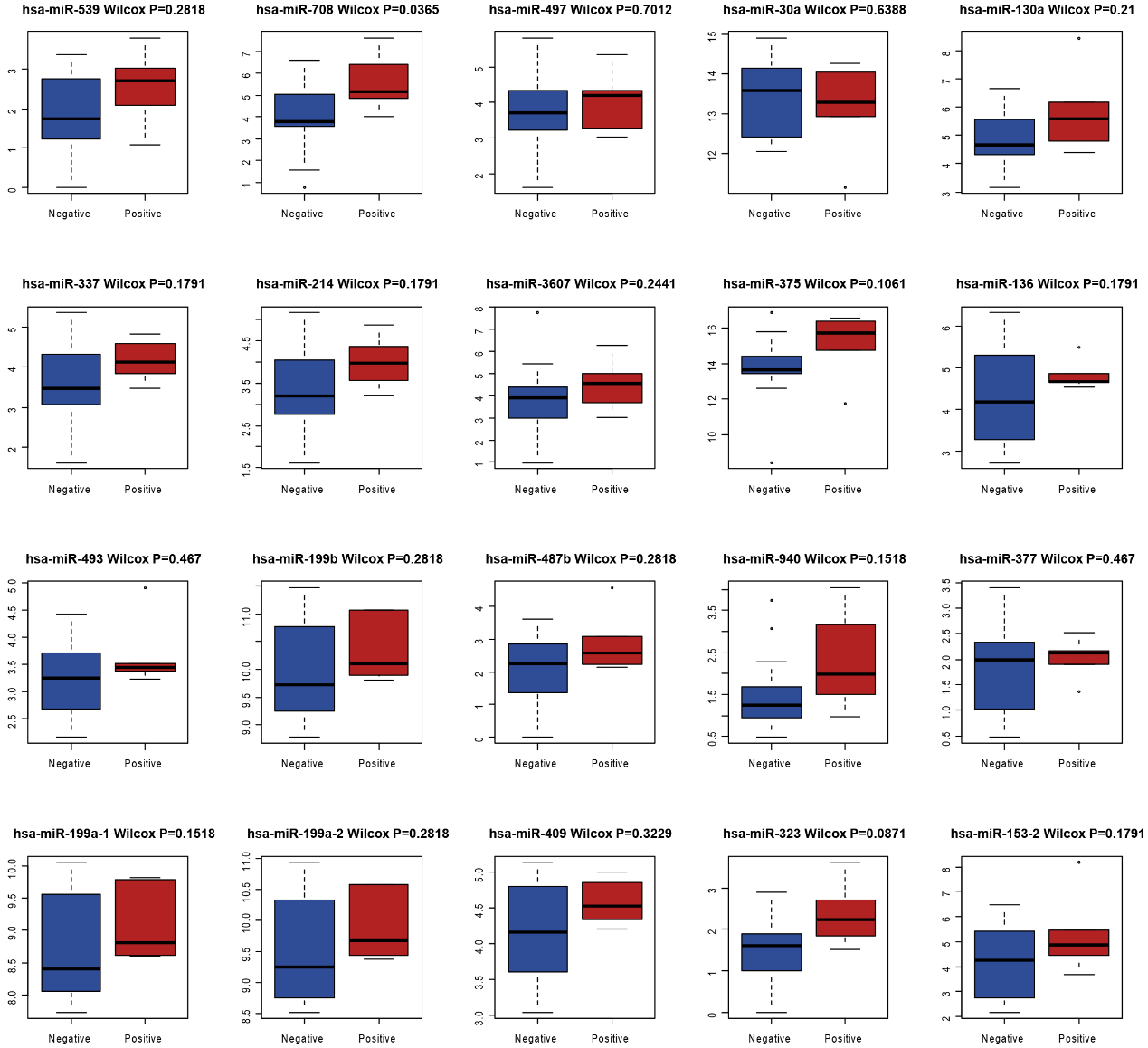
**

**Supplemental Figure S1. In silico validation using TCGA database.** Expression levels of each miRNA (miR-539, miR-708, miR-497, miR-30a, miR-130a, miR-337, miR-214, miR-3607, miR-375, miR-136, miR-493, miR-199b, miR-487b, miR-940, miR-377, miR-199a-5p, miR-199a-3p, miR-409, miR-153-3p, and miR-323) between lymph node-negative and lymph node-positive samples in T1b patients (12 LN(-) v.s. 6 LN(+))in TCGA database.


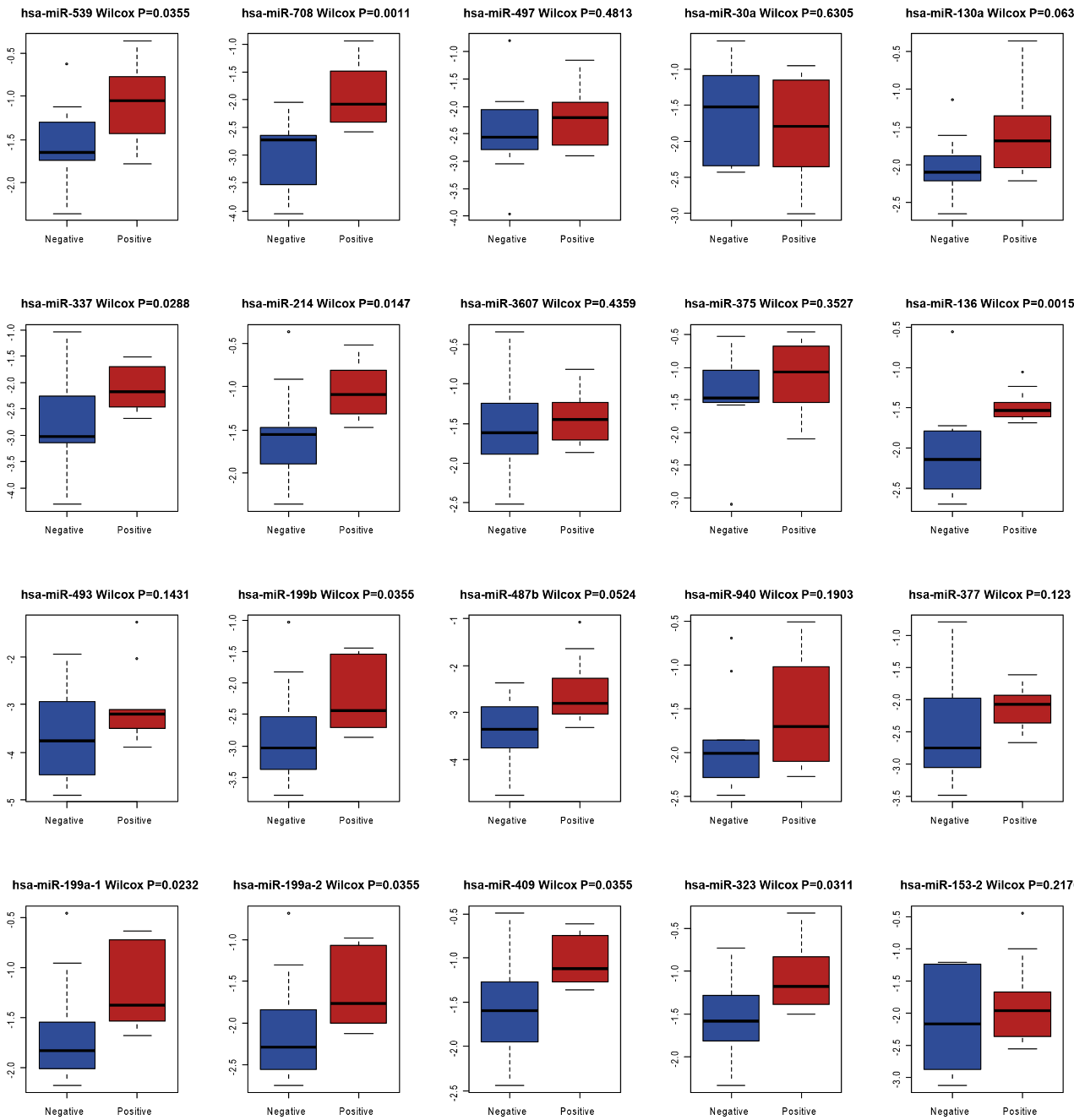


**Supplemental Figure S2. Validation using qRT-PCR.** Expression levels of each miRNA (miR-539, miR-708, miR-497, miR-30a, miR-130a, miR-337, miR-214, miR-3607, miR-375, miR-136, miR-493, miR-199b, miR-487b, miR-940, miR-377, miR-199a-5p, miR-199a-3p, miR-409, miR-153-3p, and miR-323) between lymph node-negative and lymph node-positive samples was determined with qRT-PCR in 20 patients (10 LN(-) v.s. 10LN(+)) from cohort 1.


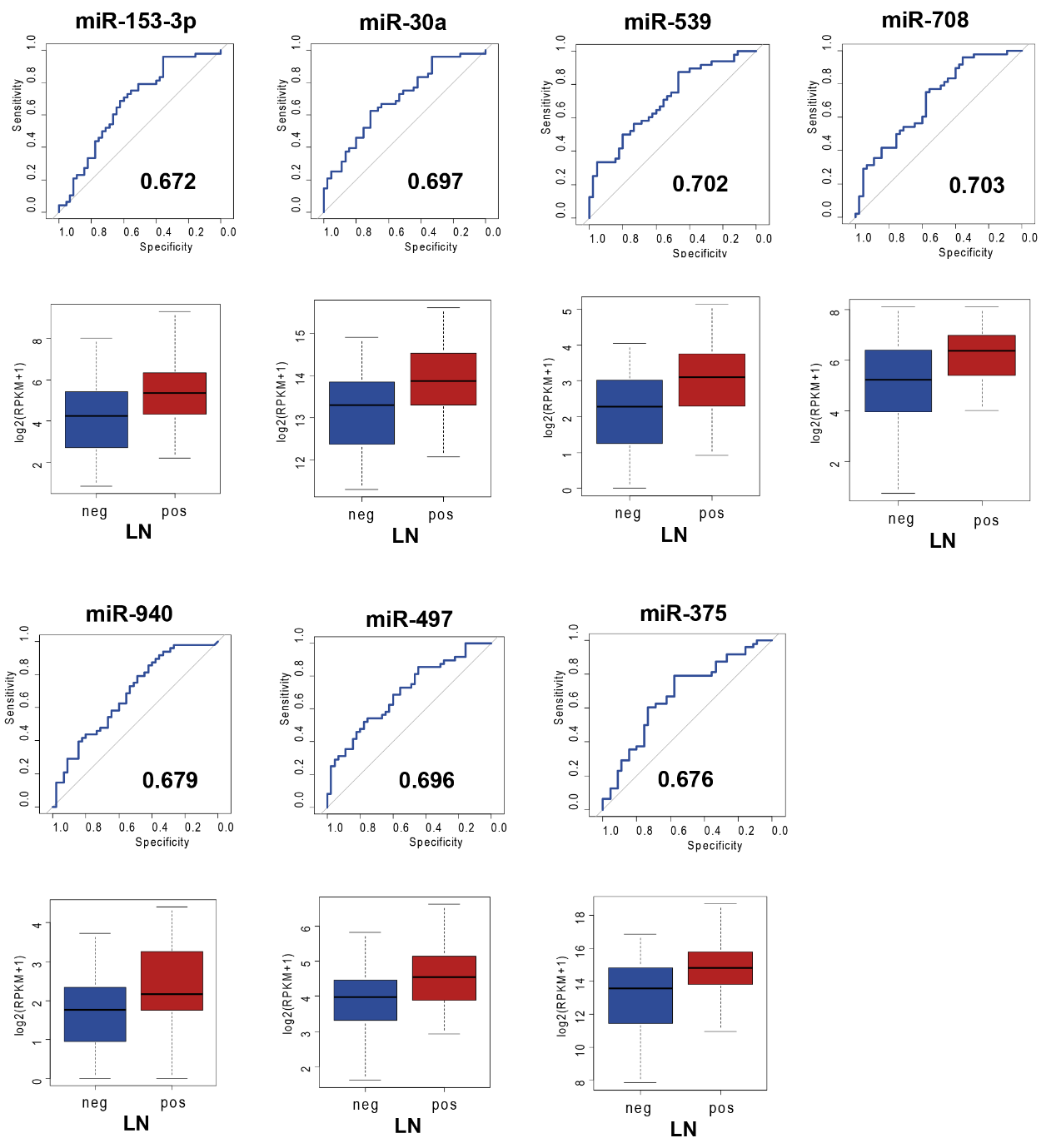


**Supplemental Figure S3. In silico validation using TCGA database.** Expression levels of each miRNA (miR-153-3p, miR-30a, miR-539, miR-708, miR-940, miR-497 and miR-375) between lymph node-negative and lymph node-positive samples and AUROCs in T1+T2 patients in TCGA database. The AUROCs of each miRNA in T1-2 patients in TCGA database.


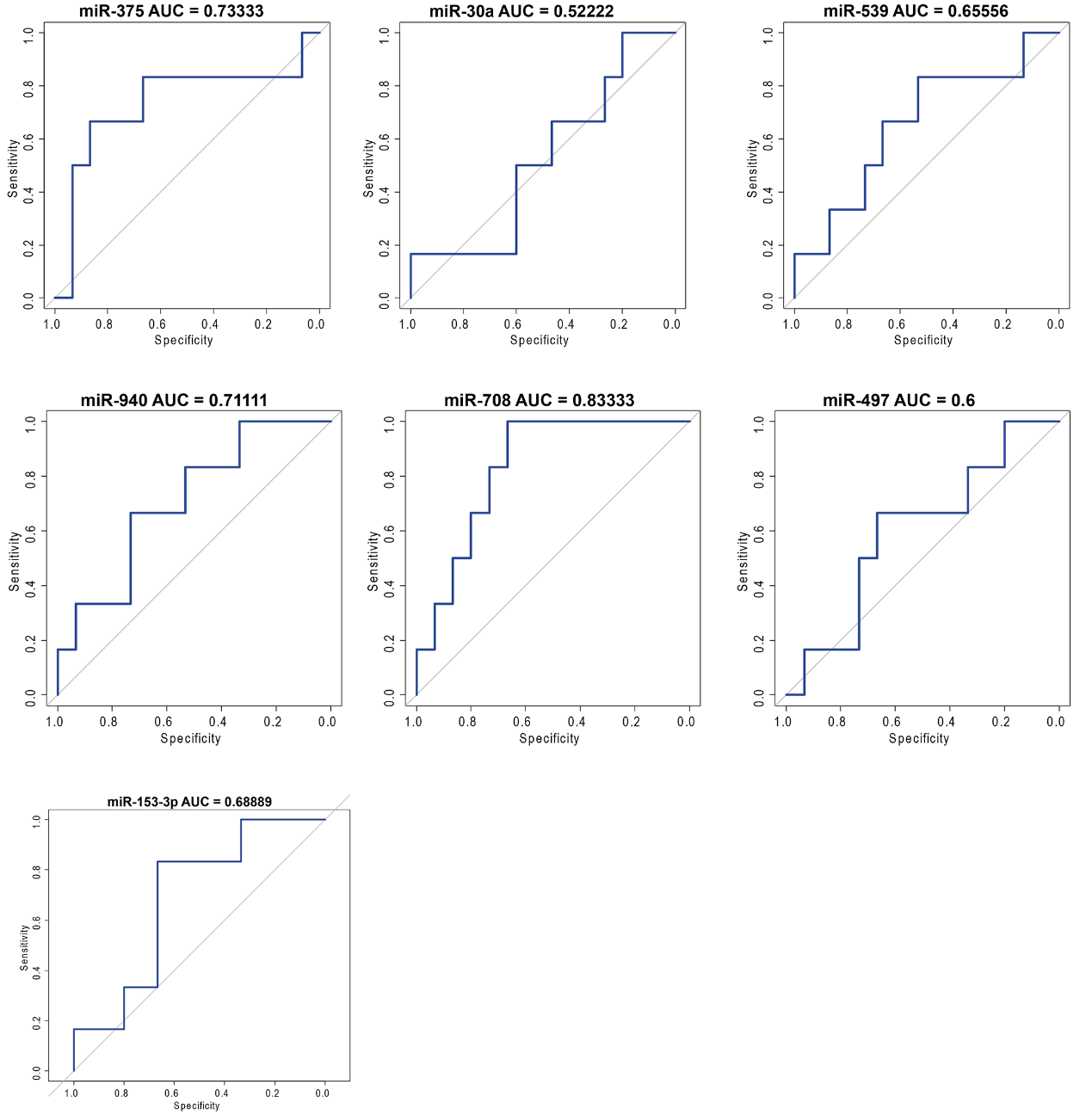


**Supplemental Figure S4.** The AUROCs of each miRNA in T1a+b patients in TCGA database.


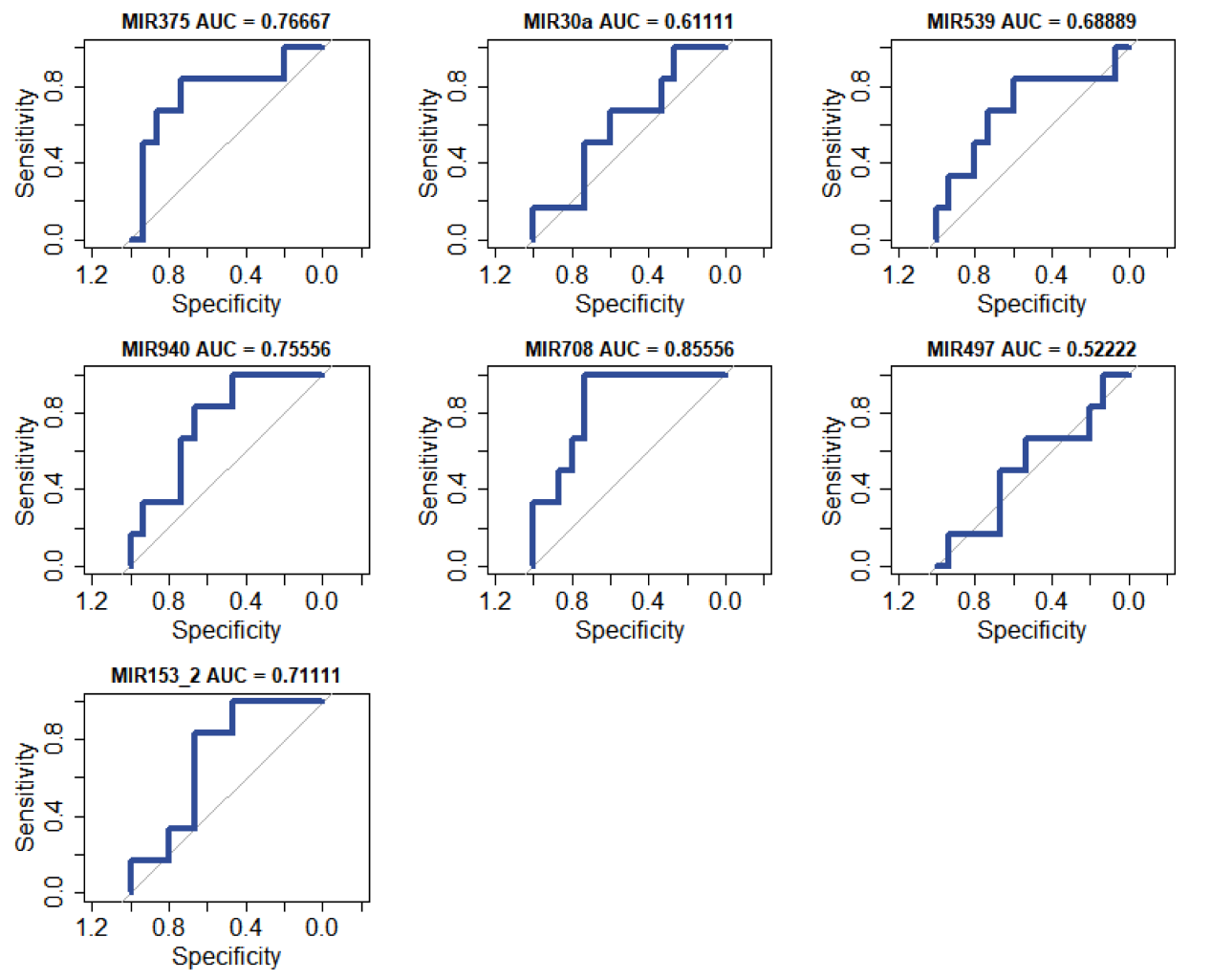


**Supplemental Figure S5.** The AUROCs of each miRNA in T1b patients in TCGA database.


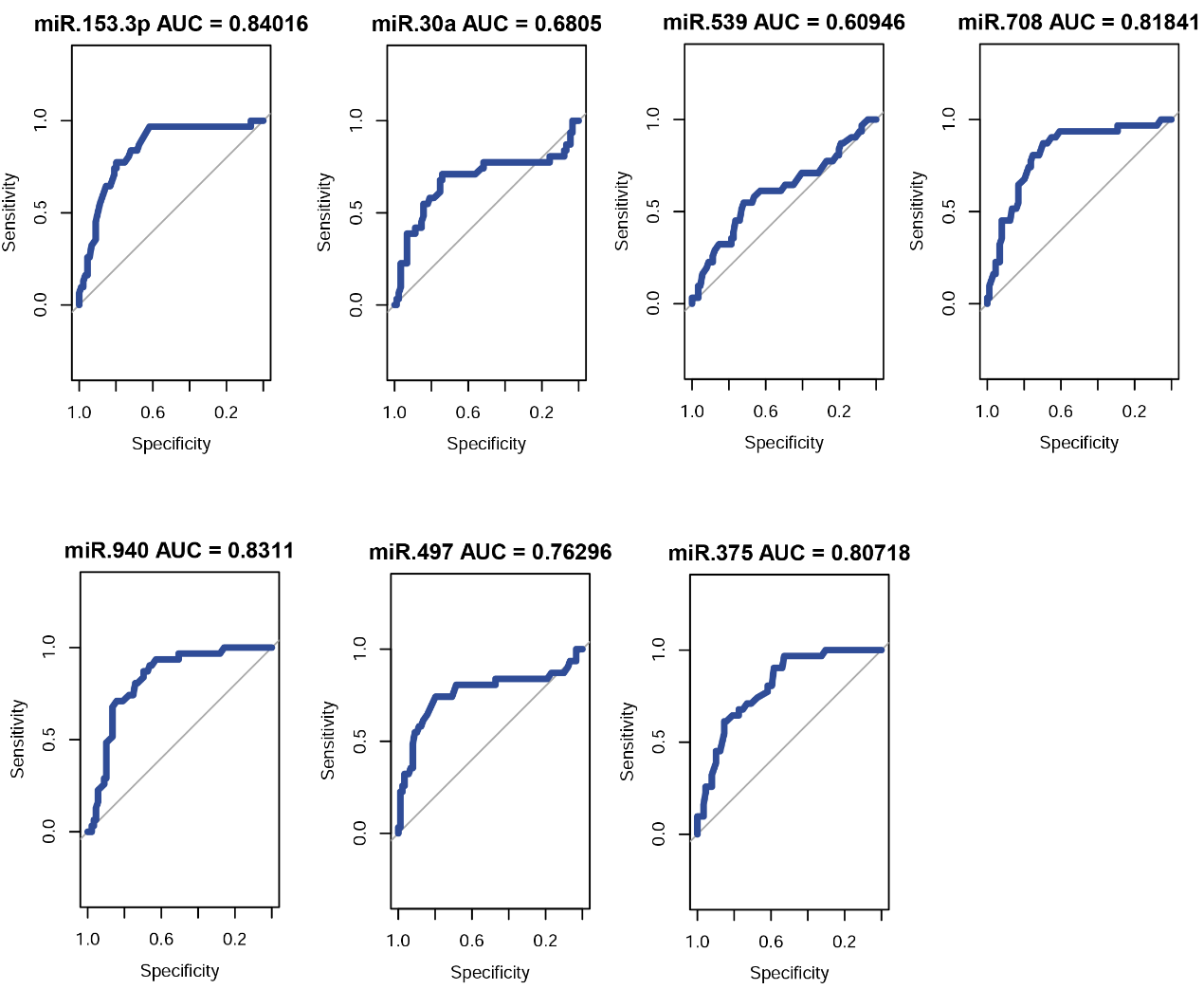


**Supplemental Figure S6.** The AUROCs of each miRNA (miR-153-3p, miR-30a, miR-539, miR-708, miR-940, miR-497 and miR-375) in T1b patients in cohort 1.

 **Supplemental Figure S7.** The survival significance of the miRNA signature in TCGA.

**Supplemental Table S1**. Associations Between Lymph Node Metastasis and Clinicopathological Features in T1b GC Patients in cohort 1 and 2.

| Characterisitcs | Cohort 1 | |  |  | Cohort 2 | |  |
| --- | --- | --- | --- | --- | --- | --- | --- |
|  | LN-negative | LN-positive | p value |  | LN-negative | LN-positive | p value |
| Gender |  |  |  |  |  |  |  |
| Male | 65 | 21 | 0.645 |  | 35 | 10 | 0.360 |
| Female | 24 | 10 |  |  | 12 | 6 |  |
| Age (years) |  |  |  |  |  |  |  |
| ≤59 | 33 | 13 | 0.671 |  | 17 | 7 | 0.767 |
| ≥60 | 56 | 18 |  |  | 30 | 9 |  |
| Tumor site |  |  |  |  |  |  |  |
| Upper third | 23 | 3 | 0.379 |  | 12 | 2 | 0.141 |
| Middle third | 9 | 5 |  |  | 7 | 1 |  |
| Lower third | 57 | 23 |  |  | 28 | 13 |  |
| Tumor depth |  |  |  |  |  |  |  |
| <500 µm | 32 | 5 | 0.044* |  | 17 | 1 | 0.026* |
| ≥500 µm | 57 | 26 |  |  | 30 | 15 |  |
| Tumor size (cm) |  |  |  |  |  |  |  |
| <3 | 66 | 19 | 0.251 |  | 34 | 10 | 0.534 |
| ≥3 | 23 | 12 |  |  | 13 | 6 |  |
| Gross type |  |  |  |  |  |  |  |
| Elevated/flat | 64 | 18 | 0.181 |  | 28 | 12 | 0.371 |
| Depressed | 25 | 13 |  |  | 19 | 4 |  |
| Histology |  |  |  |  |  |  |  |
| well and morderate | 62 | 9 | <0.001* |  | 34 | 3 | <0.001* |
| Poor | 27 | 22 |  |  | 13 | 13 |  |
| Lymphovascular tumor emboli | |  |  |  |  |  |  |
| Absent | 79 | 20 | 0.005* |  | 42 | 9 | 0.008* |
| Present | 10 | 11 |  |  | 5 | 7 |  |
| CEA |  |  |  |  |  |  |  |
| normal | 78 | 26 | 0.556 |  | 42 | 12 | 0.215 |
| high | 11 | 5 |  |  | 5 | 4 |  |
| CA19-9 |  |  |  |  |  |  |  |
| normal | 82 | 26 | 0.294 |  | 43 | 15 | 1.000 |
| high | 7 | 5 |  |  | 4 | 1 |  |
| CA72-4 |  |  |  |  |  |  |  |
| normal | 79 | 24 | 0.139 |  | 41 | 11 | 0.128 |
| high | 10 | 7 |  |  | 6 | 5 |  |
| CT diagnosis |  |  |  |  |  |  |  |
| Lymph node-negative | 65 | 19 | 0.258 |  | 35 | 9 | 0.213 |
| Lymph node-positive | 24 | 12 |  |  | 12 | 7 |  |

* *p* values <0.05 were considered

**Supplemental Table S2**. Associations Between Lymph Node Metastasis and Clinicopathological Features in T1b GC Patients in cohort 3 and 4.

| Characterisitcs | Cohort 3 | |  |  | Cohort 4 | |  |
| --- | --- | --- | --- | --- | --- | --- | --- |
|  | LN-negative | LN-positive | p value |  | LN-negative | LN-positive | p value |
| Gender |  |  |  |  |  |  |  |
| Male | 55 | 18 | 0.620 |  | 51 | 23 | 0.536 |
| Female | 25 | 6 |  |  | 25 | 15 |  |
| Age (years) |  |  |  |  |  |  |  |
| ≤59 | 29 | 8 | 1.000 |  | 43 | 24 | 0.549 |
| ≥60 | 51 | 16 |  |  | 33 | 14 |  |
| Tumor site |  |  |  |  |  |  |  |
| Upper third | 21 | 2 | 0.331 |  | 13 | 1 | 0.112 |
| Middle third | 10 | 4 |  |  | 28 | 13 |  |
| Lower third | 49 | 18 |  |  | 35 | 24 |  |
| Tumor depth |  |  |  |  |  |  |  |
| <500 µm | 30 | 4 | 0.082 |  | 27 | 16 | 0.031* |
| ≥500 µm | 50 | 20 |  |  | 49 | 32 |  |
| Tumor size (cm) |  |  |  |  |  |  |  |
| <3 | 63 | 14 | 0.063 |  | 51 | 28 | 0.524 |
| ≥3 | 17 | 10 |  |  | 25 | 10 |  |
| Gross type |  |  |  |  |  |  |  |
| Elevated/flat | 48 | 17 | 0.471 |  | 52 | 20 | 0.106 |
| Depressed | 32 | 7 |  |  | 24 | 18 |  |
| Histology |  |  |  |  |  |  |  |
| well and morderate | 55 | 5 | <0.001* |  | 46 | 15 | 0.046* |
| Poor | 25 | 19 |  |  | 30 | 23 |  |
| Lymphovascular tumor emboli | |  |  |  |  |  |  |
| Absent | 70 | 16 | 0.029* |  | 71 | 22 | <0.001* |
| Present | 10 | 8 |  |  | 5 | 16 |  |
| CEA |  |  |  |  |  |  |  |
| normal | 70 | 18 | 0.194 |  | 68 | 29 | 0.092 |
| high | 10 | 6 |  |  | 8 | 9 |  |
| CA19-9 |  |  |  |  |  |  |  |
| normal | 74 | 19 | 0.122 |  | 70 | 31 | 0.121 |
| high | 6 | 5 |  |  | 6 | 7 |  |
| CA72-4 |  |  |  |  |  |  |  |
| normal | 73 | 19 | 0.142 |  | 68 | 30 | 0.156 |
| high | 7 | 5 |  |  | 8 | 8 |  |
| CT diagnosis |  |  |  |  |  |  |  |
| Lymph node-negative | 62 | 14 | 0.072 |  | 45 | 26 | 0.414 |
| Lymph node-positive | 18 | 10 |  |  | 31 | 12 |  |

* *p* values <0.05 were considered
